# Swelling-Activated Ca^2+^ Channels Trigger Ca^2+^ Signals in Merkel Cells

**DOI:** 10.1371/journal.pone.0001750

**Published:** 2008-03-12

**Authors:** Henry Haeberle, Leigh A. Bryan, Tegy J. Vadakkan, Mary E. Dickinson, Ellen A. Lumpkin

**Affiliations:** 1 Neuroscience Graduate Program, University of California San Francisco, San Francisco, California, United States of America; 2 Department of Neuroscience, Baylor College of Medicine, Houston, Texas, United States of America; 3 Department of Molecular Physiology and Biophysics, Baylor College of Medicine, Houston, Texas, United States of America; 4 Department of Molecular and Human Genetics, Baylor College of Medicine, Houston, Texas, United States of America; Vrije Universiteit Amsterdam, Netherlands

## Abstract

Merkel cell-neurite complexes are highly sensitive touch receptors comprising epidermal Merkel cells and sensory afferents. Based on morphological and molecular studies, Merkel cells are proposed to be mechanosensory cells that signal afferents via neurotransmission; however, functional studies testing this hypothesis in intact skin have produced conflicting results. To test this model in a simplified system, we asked whether purified Merkel cells are directly activated by mechanical stimulation. Cell shape was manipulated with anisotonic solution changes and responses were monitored by Ca^2+^ imaging with fura-2. We found that hypotonic-induced cell swelling, but not hypertonic solutions, triggered cytoplasmic Ca^2+^ transients. Several lines of evidence indicate that these signals arise from swelling-activated Ca^2+^-permeable ion channels. First, transients were reversibly abolished by chelating extracellular Ca^2+^, demonstrating a requirement for Ca^2+^ influx across the plasma membrane. Second, Ca^2+^ transients were initially observed near the plasma membrane in cytoplasmic processes. Third, voltage-activated Ca^2+^ channel (VACC) antagonists reduced transients by half, suggesting that swelling-activated channels depolarize plasma membranes to activate VACCs. Finally, emptying internal Ca^2+^ stores attenuated transients by 80%, suggesting Ca^2+^ release from stores augments swelling-activated Ca^2+^ signals. To identify candidate mechanotransduction channels, we used RT-PCR to amplify ion-channel transcripts whose pharmacological profiles matched those of hypotonic-evoked Ca^2+^ signals in Merkel cells. We found 11 amplicons, including PKD1, PKD2, and TRPC1, channels previously implicated in mechanotransduction in other cells. Collectively, these results directly demonstrate that Merkel cells are activated by hypotonic-evoked swelling, identify cellular signaling mechanisms that mediate these responses, and support the hypothesis that Merkel cells contribute to touch reception in the Merkel cell-neurite complex.

## Introduction

The mechanical senses of touch, proprioception, hearing and balance are initiated by cells that transduce force into electrical signals. Though the mechanotransduction events underlying audition and balance have been extensively investigated [1], [2], little is known about the signals that initiate the somatic mechanical senses of proprioception and touch [3], [4]. Progress has been hampered by the anatomy of the somatosensory system. Mechanotransduction occurs in peripheral afferent terminals, which are heterogeneous and widely distributed throughout the body, making most somatosensory mechanoreceptors difficult to study directly.

Merkel cell-neurite complexes, which are cutaneous mechanoreceptors critical for fine shape and texture discrimination [4], have several characteristics that overcome these experimental challenges. Unlike most cutaneous touch receptors, the response patterns of these touch receptors have been identified in semi-intact recording preparations: when the skin is displaced they generate the slowly adapting type I (SAI) response [5], [6]. Furthermore, in transgenic *Math1/nGFP* mice, Merkel cells are specifically labeled with green fluorescent protein (GFP) [7], permitting enrichment of Merkel cells from dissociated skin using fluorescence-activated cell sorting (FACS) [8].

Since their discovery, epidermal Merkel cells have been proposed to be mechanosensory cells that transduce mechanical stimuli and then transmit sensory information to underlying sensory afferents [9]. This hypothesis stems from their location adjacent to sensory afferent terminals in highly touch sensitive areas of skin. This anatomy resembles that of hair cells, the force-sensitive epithelial cells of the inner ear that make synaptic contacts with afferent terminals. Moreover, Merkel cells express presynaptic molecules essential for synaptic transmission [8], [10], [11]. Finally, Merkel cells have functional VACCs [8], [12], which trigger vesicle release at neuronal synapses. Despite this evidence, physiological experiments have failed to conclusively determine if Merkel cells are required for the SAI response [13]. Eliminating Merkel cells by laser ablation or genetic deficiency abolished the SAI response in some studies [14], [15] but not others [16], [17]. Previous reports exploring Merkel-cell mechanosensitivity *in vivo* and *in vitro* have been likewise inconclusive [18], [19].

To determine if Merkel cells are mechanosensory cells, we asked if purified Merkel cells directly respond to mechanical stimuli *in vitro*. When the skin is displaced, forces must alter Merkel-cell shape, though the exact nature of this deformation is not known. To induce such a shape change *in vitro*, we chose to stimulate Merkel cells with osmotic stimuli because they permit simultaneous stimulation of many cells. Also, this is a robust stimulus that activates force-sensitive ion channels [20]–[22] as well as mechanosensitive cells such as hair cells and somatosensory neurons [23], [24]. Moreover, MscL, the mechanosensitive channel whose structure and function is best understood, is directly activated by hypotonic stimuli in native cells [25]. Our findings demonstrate that hypotonic stimuli cause Ca^2+^ influx in purified Merkel cells, and indicate that this Ca^2+^ influx is initiated by swelling-activated ion channels. We used RT-PCR and pharmacology to identify candidate ion channels that may mediate this response. Our results demonstrate that Merkel cells are directly activated by a mechanical stimulus, which supports the hypothesis that they function as mechanoreceptors in Merkel cell-neurite complexes.

## Materials and Methods

### Cell preparation

All animal research was conducted according to protocols approved by Institutional Animal Care and Use Committee (IACUC) of Baylor College of Medicine (BCM) and the University of California, San Francisco (UCSF). Merkel cells were dissociated from the skin of postnatal day 3–6 (P3–P6) *Math1/nGFP* [7], [8] mice after euthanasia by decapitation with sharp scissors. The skin from the body and face was dissected and washed in 10% Hibiclens (Regent Medical) and Hank's balanced salt solution (HBSS) supplemented with penicillin, streptomycin and amphotericin B. Tissue was cut into 1-cm^2^ pieces and incubated for 1 h at 23°C in dispase (BD Biosciences) suspended to 25 U/mL in Ca^2+^ and Mg^2+^ free HBSS. The epidermis was peeled from the dermis with sharp forceps and incubated in 0.1% trypsin and 1 mM EDTA-4Na solution (Gibco) for 15 min with periodic vortexing. Trypsin was neutralized with fetal bovine serum (FBS) and cells were triturated with a 5-ml serological pipette. Cells were filtered with 70- and 40-µm cell strainers, spun at 400×*g* for 12–15 min and then resuspended in keratinocyte media (CNT-02, Chemicon) with 10% FBS. GFP-positive Merkel cells were enriched to approximately 85% by FACS into a landing media containing 50% FBS and 50% keratinocyte media (CNT-02, Chemicon). Merkel cells were spotted onto either collagen-coated coverslips for Ca^2+^ imaging or collagen-coated eight-well chamber slides (LAB-TEK) for cell-volume analysis and grown with 5% CO_2_ at 37°C in antibiotic-free keratinocyte media (CNT-02, Chemicon).

### Live-cell Ca^2+^ imaging

After 2 days in culture, Merkel cells were loaded for 20 min with 2 µM fura-2 acetoxymethyl ester (Molecular Probes) and 0.02% pluronic F-127 (Molecular Probes) in a modified Ringer's solution containing (in mM): 110 NaCl, 5 KCl, 10 HEPES (pH 7.4), 10 D-Glucose, 2 MgCl_2_, 2 CaCl_2_ and 30 mannitol (290 mmol·kg^−1^). Cells were allowed to digest the ester bonds for 30 min and were imaged in modified Ringer's solution. Twenty percent hypotonic solutions contained all the same elements as modified Ringer's solution except mannitol. At these concentrations, mannitol did not fluoresce significantly in the fura-2 fluorescence spectra. To make 30% hypertonic solution, modified Ringer's solution was supplemented with an additional 45 mM mannitol (377 mmol·kg^−1^). For dose-response experiments, solutions contained (in mM): 95 NaCl, 5 KCl, 10 HEPES (pH 7.4), 10 D-Glucose, 2 MgCl_2_, 2 CaCl_2_ and 45 mannitol (isotonic, 290 mmol·kg^−1^); 30 mannitol (10% hypotonic, 261 mmol·kg^−1^); 15 mannitol (20% hypotonic, 232 mmol·kg^−1^) or no mannitol (30% hypotonic, 203 mmol·kg^−1^). Osmolality of all solutions was verified to be within 1% of target values with a vapor pressure osmometer (Vapro 5520, Wescor). Merkel cells were depolarized with high-K^+^ Ringer's solution containing (in mM): 70 NaCl, 75 KCl, 10 HEPES (pH 7.4), 10 D-glucose, 2 MgCl_2_ and 2 CaCl_2_.

Cells were viewed with a BX61WI epiflourescence upright microscope (Olympus) equipped with XLUMPlanFl 20×, 0.95 NA and 60×, 0.9 NA dipping objective lenses. Cells were illuminated with a 300-W Xenon lamp equipped with a high-speed excitation filter wheel (Sutter). Emission was captured with a cooled CCD camera (Hamamatsu). Data were acquired with Metafluor software of Meta Imaging series (version 6.4.7, Molecular Devices), and analyzed with custom algorithms written in Igor Pro (Version 5.03, Wavemetrics).

The Ca^2+^ dissociation constant of fura-2 in Merkel cells was determined by performing a three point calibration *in situ* with solutions containing (in mM): 135 KCl, 2 MgCl_2_, 10 HEPES, and one of the following: 10 EGTA (for R_min_), 10 CaCl_2_ (for R_max_), and 8.5 EGTA plus 1.5 CaCl_2_ (for R_mid_, effective free [Ca^2+^] = 0.9 µM as measured *in vitro* by fura-2 imaging). Merkel cells were rendered Ca^2+^-permeable by ionomycin (1 µM) and triton X-100 (0.01–0.015%). Cellular respiration was inhibited with 2 mM 2-deoxy-D-glucose to block active pumps. Only cells with stable F_340_/F_380_ and fura-2 concentrations were considered to be clamped at extracellular [Ca^2+^] and included in the analysis. Resting [Ca^2+^] ranged from 40–150 nM in healthy Merkel cells, consistent with resting [Ca^2+^] in sensory cells [26], [27].

### Pharmacology

Unless noted, reagents were purchased from Sigma. To assess pharmacological sensitivity of hypotonic-evoked signals, Merkel cells were bathed in modified Ringer's solution containing either 10 µM Ruthenium Red or 50 µM amiloride. At higher concentrations, the fluorescence of amiloride obscured fura-2 fluorescence signals. L-, P/Q- and N-type VACCs were blocked by a mixture of 10 µM nimodipine (Tocris) and 10 µM ω-conotoxin MVII-C (Tocris) [8]. Internal Ca^2+^ stores were depleted by application of 1 µM thapsigargin (Tocris) followed by repeated high-K^+^ pulses to activate store release.

### Volume imaging and analysis

Merkel cells were cultured for two days in an eight-well coverglass chamber (Lab-Tek). Cells were imaged in modified Ringer's solution described above containing 0.1-µm fluorescent microspheres (TetraSpeck, Invitrogen). Microspheres were allowed to settle onto the surfaces of Merkel cells and coverslips for 20–30 min. Merkel cells were imaged with an LSM 5-LIVE imaging system with a Plan-Apocromat 63×, 1.4 NA oil-immersion objective lens (Zeiss). Microsphere fluorescence was excited at 532 nm, and epifluorescence emission passed through a 535-nm dichroic beam splitter and a 550-nm long-pass filter. Stacks of confocal sections were imaged once every 7 s. Microsphere locations were determined with the “spot” identifying utility in Imaris 5.0.1 software (Bitplane AG). Volume calculations and graphs were generated in custom programs written for MATLAB (Mathworks).

### Confocal imaging of Merkel-cell morphology

Cells were imaged in a modified Ringer's solution described above containing 1 µM BODIPY FL C5-ceramide (Molecular Probes) and 0.02% pluronic F-127 (Molecular Probes). Fluorescent sphingolipids were allowed to diffuse into cell membranes for 5 min and then were imaged with the system described for volume imaging.

### RT-PCR

GFP^+^ Merkel cells were purified from P3–P6 mice using FACS with strict gating conditions to achieve ≥95% purity. Cell from two to six mice were used for each sort. Total RNA from 2–10×10^4^ GFP^+^ cells was isolated using commercially available reagents (Qiagen RNeasy kit) and DNAse treated according to manufacturer's instructions to remove contaminating genomic DNA. First-strand cDNA was synthesized using oligo(dT)_12–18_ primers at 42°C for 2 h with SuperScriptIII (Invitrogen). PCR products were amplified with touchdown PCR using a PTC-200 Peltier thermal cycler (MJ Research); cDNA from ∼1000 cells was used for each reaction. To evaluate reproducibility, each primer pair was tested on two to four independent biological samples, that is, cDNA produced from cells purified in separate experiments. We considered amplicons robust if they were present in at least half of biological samples tested. In all experiments, control PCRs lacking cDNA template were performed to confirm the absence of contamination, and primer performance was verified with positive control cDNA template from brain, skin, or a mixture of liver, heart, spleen and kidney tissue. With the exception of PKD-REJ, a gene predicted to have no introns (Entrez Gene accession number NM_011105), primer pairs were designed to span introns to ensure that amplicons were not derived from genomic DNA.

### Statistical analysis

A Merkel cell was considered unhealthy and excluded from analysis if (1) its resting [Ca^2+^] was 30% higher than the mean of all cells in the experiment, (2) its [Ca^2+^] remained elevated post stimulus. Cells were considered responsive to stimulation if [Ca^2+^] increased above threshold, as defined as 3 •Δ+*x̄*. Δ is the range of values recorded during 30 s of imaging under control conditions, and *x̄* is the mean of control values. Response latency was defined as the amount of time elapsed between solution change and the time when cellular [Ca^2+^] first exceeded threshold. Because peak hypotonic-evoked Ca^2+^ signals in individual experiments were not normally distributed, we used the non-parametric Wilcoxon signed rank test to analyze cellular responses in individual experiments. Mean peak hypotonic-induced responses of cells in individual wells were near-normally distributed (*N* = 20 means, skewness = 0.35, excess kurtosis = 0.75, *N* = 7–28 cells per experiment); therefore, we used paired Student's *t* tests to compare paired mean responses.

## Results

### Hypotonic stimuli evoke cytoplasmic Ca^2+^ signals in Merkel cells

**Figure 1:**
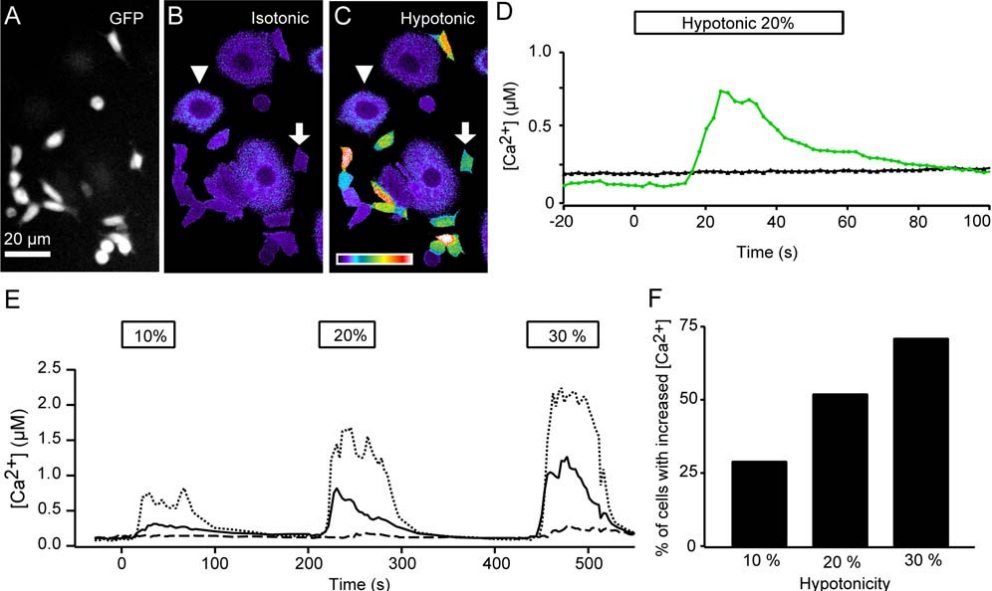
GFP^+^ Merkel cells but not keratinocytes show cytoplasmic [Ca^2+^] increases in response to hypotonic solutions. (A) An epiflourescence micrograph shows GFP^+^ Merkel cells after two days in culture (scale bar: 20 µm). (B, C) Pseudocolor images of fura-2 fluorescence ratios (F_340_/F_380_) of cells just before perfusion (B) with a 20% hypotonic solution (232 mmol·kg^−1^), and 26 s after perfusion onset (C). Pseudocolor scale bar represents F_340_/F_380_ (range 0.3–3.0). (D) Plot of [Ca^2+^] in a keratinocyte (denoted by arrowhead in C, black) and a Merkel cell (arrow in C, green). (E) Plot of cytoplasmic [Ca^2+^] versus time for three representative Merkel cells perfused with bath solutions of decreasing osmolality. (F) Quantification of the proportion of total Merkel cells (*N* = 104) that responded to hypotonic solutions (see Methods, 10%: 263 mmol·kg^−1^; 20%: 232 mmol·kg^−1^: 30%: 203 mmol·kg^−1^).

In cells lacking cell walls, hypotonic extracellular solutions cause water flux across the cell membrane to induce cell swelling. If Merkel cells express ion channels activated by membrane tension, we reasoned that cell swelling might increase membrane tension to open such channels. The resulting membrane depolarization could then activate VACCs to allow Ca^2+^ influx. To determine if Merkel cells respond to changes in osmolality, we monitored intracellular Ca^2+^ with the ratiometric, fluorescent indicator fura-2. In epidermal-cell suspensions, Merkel cells represent ≈0.2% of dissociated cells. Using FACS we enriched GFP^+^ Merkel cells to approximately 85% percent: the remaining 15% consisted predominately of GFP-negative keratinocytes. Cells were subjected to Ringer's solutions of varying osmolality. Most Merkel cells showed an increase in free [Ca^2+^] in response to 20% hypotonic stimuli (65±3% cells, *N* = 19 experiments, 10–33 cells/experiment, Fig. 1A–C). In responding Merkel cells, the peak cytoplasmic Ca^2+^ transients ranged from threshold to 4 µM above resting Ca^2+ ^levels (0.51±0.06 µM, mean±SEM, *N* = 19 experiments, 47–90% of cells responded per experiment). By contrast, Merkel cells showed no changes in cytoplasmic [Ca^2+^] in 30% hypertonic solutions (*N* = 35 cells). Keratinocytes showed no change in free [Ca^2+^] to osmotic strength changes (*N* = 30 cells, Fig. 1A–C, arrow head). Our results indicate that Merkel cells, but not keratinocytes, respond to hypotonic solutions with increased cytoplasmic [Ca^2+^].

We next characterized the time course of the hypotonic response in Merkel cells. Hypotonic solutions triggered Ca^2+^ transients with an initial rise in global [Ca^2+^] 16.4±1.5 s after onset of perfusion (Fig. 1D, *N* = 19 experiments, 7–28 cells/experiment). In control experiments with fluorescent perfusion solutions, we that ≥95% of the bath volume was replaced in 5.5 s. Subtracting this perfusion latency from Merkel cells' response times indicates that 10.9±1.5 s elapsed before the initial rise in global [Ca^2+^] during hypotonic stimulation. Merkel cells responded to hypotonic Ringer's solution with a sigmoidal increase in [Ca^2+^]. In many cells, Ca^2+^ signals reached a plateau, then decayed exponentially to an elevated [Ca^2+^] significantly above baseline (*p*<0.0001, *N* = 68 cells, paired Wilcoxon test). After the hypotonic stimulus ended, cytoplasmic free [Ca^2+^] recovered to baseline. The time course of recovery was fit with a single exponential with a time constant of 22±2.6 s (mean±SD; *N* = 68 cells).

To characterize the dose-response of hypotonic-triggered Ca^2+^ increases in Merkel cells, we challenged Merkel cells with solutions of progressively decreasing osmolality. Ringer's solutions of decreasing osmolality induced larger peak Ca^2+^ transients in individual Merkel cells (Fig. 1E). Merkel cells with the largest Ca^2+^ transients at 10% hypotonic Ringer's solution also had the largest Ca^2+^ increase at 20 or 30% hypotonic Ringer's solution. In addition, solutions of progressively lower osmotic strength elicited responses in a greater proportion of Merkel cells than mildly hypotonic solutions (261 mmol·kg^−1^ recruited 29% of cells, 232 mmol·kg^−1^ recruited 52% of cells, 203 mmol·kg^−1^ recruited 71% of cells, Fig. 1F). Our data indicate that Merkel cells respond to relatively mild 10% changes in osmolality, yet, even 30% hypotonic solutions do not appear to saturate Merkel cells' responses.

**Figure 2:**
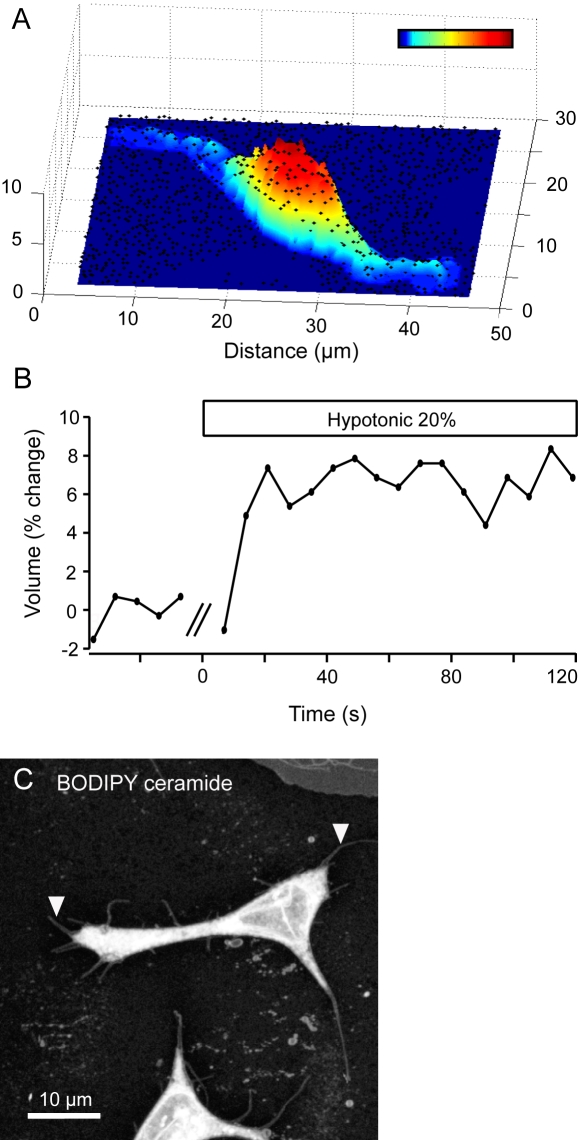
Merkel cells swell in hypotonic solutions. Shape changes were monitored with plasmalemma-bound fluorescent microspheres using confocal microscopy. (A) A three-dimensional plot of fluorescent microspheres (black spots) coating the surfaces of a representative Merkel cell and the coverslip. The surface of the Merkel cell was reconstructed from the location of the micropheres (topographic scalebar: navy blue denotes the coverslip surface; dark red = 6 µm above the coverslip). Areas of low bead density are keratinocytes, which are poorly bound by the beads. (B) Plot of cell volume versus time for the cell shown in (A). The 20% hypotonic solution was administered at t = 0. Double slash marks denote a transient focal-plane change during the solution change. (C) Projections of confocal z-series of cells membranes stained with fluorescent sphingolipids. Processes 1–8 µm in length jutted from Merkel cells' surfaces (arrowheads). By comparison, a keratinocyte showed a smooth cell surface (upper right).

Hypotonic solutions induce cell swelling that activates stretch-sensitive channels in bacteria [28], so we asked whether similar hypotonic-induced swelling occurred in Merkel cells. To ascertain if hypotonic solutions altered Merkel-cell volume, we monitored cell shape in three dimensions with fluorescent microspheres attached to the plasmalemma of Merkel cells while perfusing cells with a 20% hypotonic bath solution. Microspheres settled onto Merkel cells and the surrounding coverslip within 30 min of bath application and remained tightly coupled during solution changes (Fig. 2A, movie S1). Microspheres were imaged with high-speed confocal microscopy, and their positions were used to model the location of Merkel-cell surfaces in relation to the coverslip. By integrating the volume between reconstructed cell surfaces and the coverslip, we estimated that Merkel cells' volume in isotonic Ringer's solution was 334±39 µm^3^ (mean±SD, *N* = 8). To determine if Merkel cells swelled in response to a hypotonic stimulus, we imaged Merkel cells in time series while perfusing with 20% hypotonic Ringer's solution (Fig. 2B). In this condition, Merkel-cell volume was significantly higher (358±43 µm^3^, mean±SD, *N* = 8, p<0.001, paired Student's *t* test), which represents an average volume increase of 7.3±2.9%. Merkel cells began swelling within 7 s of the onset of hypotonic perfusion, which was the temporal resolution of the three-dimensional imaging. Merkel cells remained enlarged during 120 s of hypotonic stimulation and relaxed to their original volume after perfusion of isotonic Ringer's solution. One Merkel cell subjected to prolonged hypotonic stimulation showed volume decreases in ∼300 s.

### Hypotonic-induced Ca^2+^ transients are concentrated in processes

**Figure 3:**
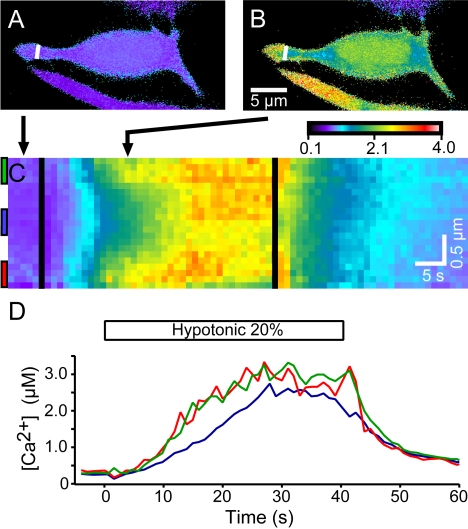
Merkel cells display increased [Ca^2+^] near the plasmalemma in response to hypotonic stimuli. (A, B) Pseudocolor images of fura-2 fluorescence ratios in a Merkel cell before (A) and 16 s after (B) perfusion onset with a 20% hypotonic solution (232 mmol·kg^−1^). Pseudocolor scale bar shows F_340_/F_380_ and applies to (A–C). (C) Pseudocolor kymograph of the line shown in (A) and (B). Each pixel along the ordinate corresponds to a point on the line shown in (A) and (B). Pixels near the top and bottom of the kymograph show F_340_/F_380_ near the plasmalemma, whereas pixels in the middle correspond to the interior of the process. Time proceeds along the abscissa. The time points of (A) and (B) are indicated by arrows pointing to the kymograph in (C). Black lines denote the beginning and end of hypotonic perfusion. (D) Plot of [Ca^2+^] versus time for three positions along the cell process. Colored lines indicate the average of the corresponding colored box in (C). Red and green traces signify regions near the plasmalemma, whereas blue represents a region deeper in the cytoplasm.

Mechanosensitive ion channels are often located within specialized cellular processes that are thought to leverage forces to the channels. Hair cells have mechanoelectrical transduction channels near the tips of modified microvilli called stereocilia [29], and kidney cells detect fluid flow with mechanosensitive channels located in cilia [30]. Similarly, Merkel cells *in vivo* have actin-filled processes that penetrate overlying keratinocytes [5], [31]. To determine if Merkel cells extend cytoplasmic processes *in vitro*, we stained Merkel cells with fluorescent sphingolipids to visualize membrane morphology (Fig. 2C). We found that most cultured Merkel cells have processes arranged in a branch-like pattern, with numerous fine protrusions (1–8 µm in length) at the terminals of large processes (2–15 µm in length). Although the larger cytoplasmic processes were visible in fura-2 fluorescence images, fine protrusions were not (Figs. 1, 3).

During hypotonic stimuli, elevated [Ca^2+^] was evident in Merkel-cell cytoplasmic processes and around nuclei (Fig. 3A, B, movie S2). In cytoplasmic processes, [Ca^2+^] increased first in regions adjacent to the plasmalemma and then in regions located deeper within the cytoplasm (Fig. 3C, D). Juxtamembrane regions had higher [Ca^2+^] than interior regions during the first 30 s of hypotonic stimulation; however, both regions displayed similar peak [Ca^2+^] and similar time courses of relaxation. The increase in [Ca^2+^] near the plasmalemma of Merkel cells implies Ca^2+^ influx across the cell membrane.

### Hypotonic-induced Ca^2+^ transients are augmented by VACCs and release from internal stores

**Figure 4:**
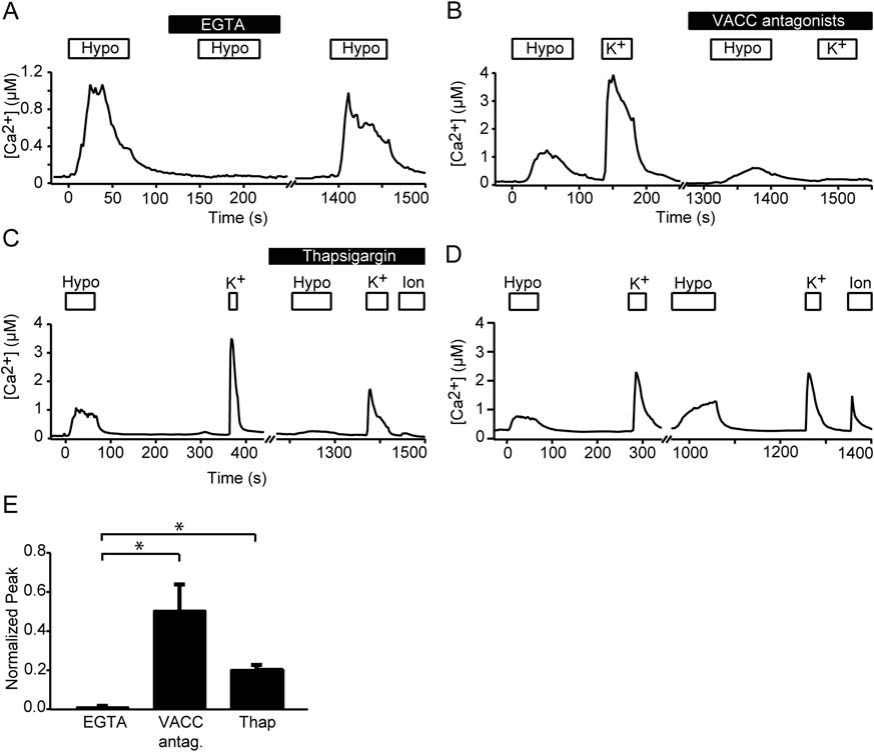
Extracellular Ca^2+ ^is required for hypotonic-triggered Ca^2+^ transients. These Ca^2+^ transients are composed in part by Ca^2+^ influx through voltage-activated Ca^2+^ channels and Ca^2+^ released from internal stores. (A–C) Plots depict [Ca^2+^] versus time of representative cells. “Hypo” denotes perfusion of 20% hypotonic solution (232 mmol·kg^−1^). (A) Labeled boxes indicate the time period of perfusion. Bath Ca^2+^ was replaced with 10 mM EGTA. (B) Cells were depolarized with high-K^+^ solution (140 mM K^+^). The cocktail of VACC antagonists contained 10 µM nimodipine and 10 µM ω-conotoxin MVII-C. (C) Thapsigargin was used at 1 µM. The box labeled “Ion” indicates a 30-s wash with 10 mM EGTA, followed by 3 minute perfusion of 10 µM ionomycin, a Ca^2+^ ionophore. (D) Same protocol as in C, but without thapsigargin treatment. (E) Quantification of the effects of EGTA, VACC antagonists and thapsigargin upon peak hypotonic-induced Ca^2+^ influx (*N* = 3–5 experiments per group). Hypotonic responses of Merkel cells exposed to these compounds were normalized to control hypotonic responses. Error bars indicate SEM. Asterisks denote statistically significant differences between EGTA and VACC or thapsigargin treated responses (p≤0.04, paired Student's *t* test). The un-normalized EGTA, VACC and thapsigargin treated responses were significantly different from their respective controls (p≤0.05, Student's *t* test).

To identify the source of hypotonic-triggered increases in cytoplasmic [Ca^2+^], we treated Merkel cells with hypotonic Ringer's solution while either blocking Ca^2+^ influx across the cell membrane or eliminating Ca^2+^ release from internal stores. We blocked Ca^2+^ influx across the membrane by transiently chelating extracellular Ca^2+^ with 10 mM EGTA. Extracellular EGTA abolished hypotonic-induced Ca^2+^ increases (99.0±0.3%, mean±SEM, *N* = 3 experiments, Fig. 4A, E), which recovered upon reintroduction of external Ca^2+^. These data indicate that extracellular Ca^2+^ influx is required for hypotonic-triggered Ca^2+^ transients.

To ascertain whether VACCs also contribute to hypotonic-induced increases in free [Ca^2+^], we blocked these channels with a cocktail containing 10 µM conotoxin MVIIC to block N- and P/Q-type Ca^2+^ channels and 10 µM nimodipine to block L-type Ca^2+^ channels [8]. The efficacy of this cocktail was tested by depolarizing Merkel cells with high-K^+^ Ringer's solution (Fig. 4B). The blocking cocktail inhibited peak high-K^+^-induced Ca^2+^ transients by 96±1% (mean±SEM, *N* = 4 experiments). By contrast, the VACC-inhibitor cocktail only curtailed peak hypotonic-induced Ca^2+^ transients by 51±13% (mean±SEM, *N* = 4 experiments). Thus, in the presence of VGCC blockers, 60% of Merkel cells had larger hypotonic-induced transients than high-K^+^ induced transients (Fig. 4B). This partial inhibition of hypotonic-evoked Ca^2+^ signals by VACC blockers indicates that VACCs contribute to, but are not the sole source of, hypotonic-induced Ca^2+^ influx across the plasma membrane.

To determine whether internal Ca^2+^ stores add to the hypotonic-induced Ca^2+^ increases, we designed an experimental protocol to empty internal Ca^2+^ stores (Fig. 4C, D). We blocked Ca^2+^ reuptake into internal stores with 1 µM thapsigargin, which inhibits the sarco/endoplasmic reticulum Ca^2+^-ATPase. As expected, Merkel cells displayed an increase in cytoplasmic Ca^2+ ^upon thapsigargin treatment (data not shown and Piskorowski *et al.*, submitted). To ensure that Ca^2+^ stores were depleted after thapsigargin treatment, we depolarized cells with high-K^+^ Ringer's solution to activate Ca^2+^-induced Ca^2+^-release. Store depletion was verified at the end of experiments by chelating extracellular Ca^2+^ with EGTA and permeabilizing cell membranes with the Ca^2+^ ionophore ionomycin. In the absence of thapsigargin, ionomycin elicited cytoplasmic Ca^2+^ transients (Fig. 4D), demonstrating that Merkel cells have detectable Ca^2+^ stores. By contrast, ionomycin-induced transients were reduced by 92±1% in cell pretreated with thapsigargin (Fig. 4C, mean±SEM, *N* = 31 cells). Pre-treatment with thapsigargin also reduced Ca^2+^ transients evoked by hypotonic stimuli by 80±3% (mean±SEM, *N* = 4 experiments).

Together, these data demonstrate that the amplitudes of hypotonic-induced transients in the presence of thapsigargin and VACC antagonists, though reduced, were significantly larger than those in the presence of 10 mM EGTA (p<0.05, paired Student's *t* test, *N* = 3–4 experiments, Fig. 4E). These results indicate that internal Ca^2+^ stores augment Ca^2+^ influx induced by hypotonic solutions.

### Pharmacology of hypotonic-evoked Ca^2+^ signals

**Figure 5:**
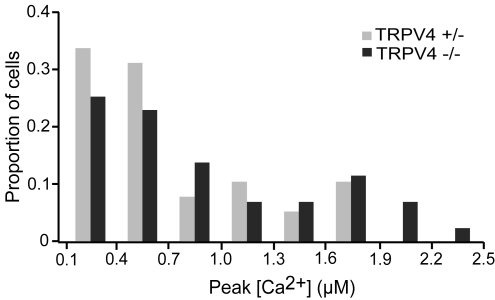
TRPV4 is not required for hypotonic-evoked [Ca^2+^] transients in Merkel cells. The paired histograms display peak osmotic responses (*N* = 37–43 cells).

Hypotonic induced extracellular Ca^2+^ influx implies the presence of a swelling-activated ion channel in the plasma membrane but does not speak to its identity. The most obvious candidate is TRPV4 (GenBank accession number NM_022017), which is expressed in Merkel cells [32] and is activated by hypotonic solutions when expressed in human embryonic kidney (HEK) cells [33], [34]. To determine if TRPV4 is required for hypotonic activation, we analyzed hypotonic-induced Ca^2+^ transients in Merkel cells from TRPV4-deficient mice (Fig. 5). The magnitude and time course of Ca^2+^ transients in Merkel cells from TRPV4 -/- mice were indistinguishable from those of heterozygous littermate controls (Fig. 5) and from wild-type responses. Thus, TRPV4 is unlikely to mediate hypotonic induced Ca^2+^ influx in Merkel cells.

We next broadened our search for the identity of swelling-activated channels in Merkel cells. Two ion-channel families have been implicated in mechanotransduction in mammals: transient receptor potential (TRP) channels and Degenerin/Epithelial Na^+^ channels (DEG/ENaC), [35]. Ruthenium red is a broad-spectrum blocker of many channels, including TRPV channels. We found that 10 µM ruthenium red inhibited peak hypotonic-induced Ca^2+^ transients by 71±8% (mean±SEM, *N* = 2 experiments, *N = *9–10 cells/experiment); however, ruthenium red inhibited high-K^+^-induced Ca^2+^ transients to the same extent (68±12%). Because ruthenium red did not fully block hypotonic-induced Ca^2+^ transients, we sought to determine if hypotonic-evoked Ca^2+^ signals were sensitive to the DEG/ENaC antagonist, amiloride. Amiloride (50 µM) did not inhibit the hypotonic-induced Ca^2+^ transient (−8±13%, *N* = 2 experiments, *N* = 20–27 cells/experiment).

### Merkel cells express TRP channels

**Figure 6:**
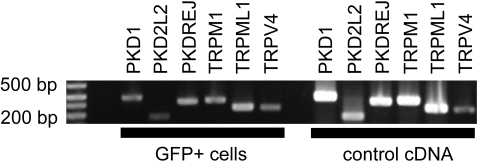
TRP channel transcripts are expressed in Merkel cells. Representative RT-PCR of the data summarized in *Table 1*. Bars at base of figure mark lanes with products amplified from GFP^+^ Merkel cells or control cDNA, respectively. Control cDNA was derived from brain or skin.

Since amiloride did not inhibit the hypotonic-evoked Ca^2+^ increases, we used RT-PCR to screen for amiloride-insensitive non-selective cation channels expressed in Merkel cells. We focused on channels from the TRP family because many of these channels fit this profile and because they function in diverse modes of sensory transduction. Primer efficacy was tested against cDNA derived from brain, skin, or a mixture of liver, skin, heart, spleen and kidney (Fig. 6). Merkel-cell cDNA yielded robust amplicons for six TRP channels and occasional amplicons for an additional five channels (Table 1). Notably, we detected amplicons for TRPC1 (GenBank accession number NM_011643), PKD1 (NM_013630) and PKD2 (NM_008861), channels previously implicated in mechanotransduction in other cell types [36], [37].

**Table 1:** RT-PCR analyses of TRP channels expressed in Merkel cells*

Gene	# indep. samples with detectable amplicons	total # indep. samples
TRPA1	0	2
**TRPC1**	**2**	**4**
TRPC2	0	2
TRPC3	0	3
TRPC4	0	3
TRPC5	0	2
TRPC6	0	3
TRPC7	0	3
**TRPM1**	**1**	**3**
TRPM2	0	3
TRPM3	0	3
TRPM4	0	2
TRPM5	0	3
TRPM6	0	3
TRPM7	0	3
TRPM8	0	2
**TRPML1**	**3**	**3**
**TRPML2**	**1**	**3**
TRPML3	0	2
**TRPP1/PKD1**	**2**	**2**
**TRPP2/PKD2**	**1**	**3**
TRPP3/PKD2L1	0	2
TRPP4/PKD1L1	0	3
**TRPP5/PKD2L2**	**2**	**2**
PKD1L2	0	2
PKD1L3	0	2
**PKD-REJ**	**2**	**3**
TRPV1	0	2
TRPV2	0	3
**TRPV3** **	**1**	**2**
**TRPV4** **	**4**	**4**
TRPV5	0	3
**TRPV6** **	**1**	**3**

* Bold font denotes transcripts with detectable expression in Merkel cells

** Expressed in Merkel cells but blocked by 10 µM ruthenium red

## Discussion

**Figure 7:**
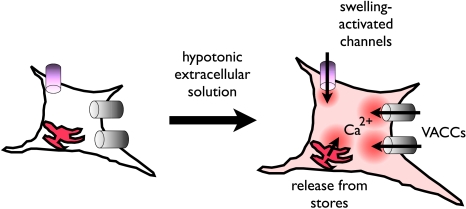
A model of hypotonic-induced Ca^2+^ transients in Merkel cells. Hypotonic solutions stimulate water influx across the cell membrane, causing cell swelling. The increased tension on the membrane activates Ca^2+^ permeable channels. These channels both contribute to a rise in Ca^2+^ influx and depolarize the membrane, opening VACCs. VACCs and Ca^2+^ release from internal stores amplify Ca^2+^ signals.

This study demonstrates that dissociated Merkel cells are directly activated by hypotonic-evoked cell swelling, and introduces a robust *in vitro* assay for analyzing the molecular mechanisms that underlie swelling-evoked signals. Based on our findings, we propose that swelling triggers Ca^2+^ entry through an as yet unknown cation channel; the resultant depolarization activates VACCs and together these two sources of Ca^2+^ influx activate Ca^2+^ release from internal stores (Fig. 7). We identified 11 TRP channels expressed in Merkel-cells, seven of which have pharmacological profiles matching the hypotonic-evoked Ca^2+^ responses we observed. Of these, PKD1 and PKD2 are promising candidates because they have been previously implicated in mechanotransduction in cilia of kidney cells [36]. Our data support a model in which skin indentation applies force to Merkel cells, whose mechanosensitive channels allow Ca^2+^ influx. This Ca^2+^ influx is augmented by VACCs and Ca^2+^ release from internal stores. These Ca^2+^ increases could trigger synaptic signaling to the underlying sensory afferent.

### Merkel cells express hypotonic-activated ion channels

Several lines of evidence indicate that Ca^2+^-permeable ion channels generate hypotonic-induced Ca^2+^ transients in Merkel cells. First, the requirement for extracellular Ca^2+^ suggests Ca^2+^ ions enter the cell across the plasma membrane. Second, blocking VACCs or emptying intracellular stores curtails, but does not eliminate, hypotonic induced Ca^2+^ increases, suggesting other sources of Ca^2+^ entry (Fig. 7). Although it is formally possible that a hypotonic-activated G-protein coupled receptor could induce membrane depolarization and Ca^2+^ release from stores, such a hypotonic-activated receptor would have to be inactivated by extracellular EGTA to explain our findings. We know of no receptors that match these requirements. Thus, the most parsimonious interpretation of our data is that hypotonic solutions induce Merkel cells to swell, causing increased membrane tension that activates Ca^2+^-permeable ion channels (Fig. 7).

Does osmosensitivity imply that Merkel cells are mechanosensory cells? Most cells have volume-regulating mechanisms that permit adaptation to osmotic changes in their environment. In addition, a variety of excitable and non-excitable cells have detectable Ca^2+^ increases in anisotonic extracellular solutions [38]–[40]. Two lines of evidence suggest hypotonic solutions might activate mechanotransduction machinery rather than ubiquitous volume regulating pathways. First, in some non-excitable cells, regulatory volume decrease is preceded by hypotonic-evoked Ca^2+^ transients with 1–5 min latencies followed by 2–10 min time to peak. By contrast, a subset of sensory cells isolated from the trigeminal nucleus has robust hypotonoic-evoked responses that develop within seconds [24]. These fast responding neurons have been proposed to be a mechanosensitive subset of trigeminal neurons. We found that hypotonic solutions induced a Ca^2+^ signal in Merkel cells similar to this rapidly activating neuronal population. Furthermore, these Ca^2+^ signals were not temporally correlated with decreases in cell volume, as would be expected for regulatory volume decreases.

The latency of hypotonic-induced Ca^2+^ influx in Merkel cells *in vitro* is ≈11 s, which is much longer than the 200 µs latency of the SAI response [41]. One explanation for this difference is that distinct molecular mechanisms may transduce hypotonic stimuli *in vitro* and touch *in vivo*. Alternatively, it is possible that the same mechanotransduction molecules are differentially activated in these two contexts. For example, Merkel cells may respond rapidly to touch-evoked pressure, whereas osmotic stimuli might take longer to develop sufficient membrane distortion to activate channels. Although our volumetric data indicate that Merkel cells begin to swell within 7 s of hypotonic-solution perfusion, it may take the observed 11 s to generate sufficient membrane tension to activate mechanotransduction channels. Alternatively, optimal gating of the Merkel cells' transduction channels *in vivo* may require extracellular linkages that are not present in culture. Extracellular links are required to transmit force to mechanotransduction channels in hair cells [35]. Moreover, mutations in specific extracellular molecules disrupt touch responses in *Caenorhabditis elegans* [42]. We postulated that hypotonic-induced membrane tension constitutes a global mechanical stimulus that is sufficient to activate force-transducing machinery in Merkel cells, but is less efficient than touch-evoked channel gating *in vivo*. The lack of extracellular linkages might also explain why direct touch failed to generate membrane currents in isolated Merkel cells in a previous report [12]. Finally, the latency of mechanotransduction is unknown in Merkel cells *in vivo*. Although the SAI response has a latency of 200 µs, it is possible that the initial phase of the SAI response is generated by the afferent and the static phase is transduced by Merkel cells [43].

*In vivo*, Merkel cells' superficial surfaces are studded with dozens of actin-rich microvilli that are proposed to be sites of mechanotransduction [9], [31]. In culture, we observed Merkel cells with two basic morphologies: cells with large processes 2–15 µm in length that branch into smaller protrusions and spherical cells with only the smaller protrusions. Both cell shapes are observed *in vivo*, where they are termed dendritic and non-dendritic Merkel cells [44]–[46]. In dendritic Merkel cells *in vitro*, we often observed hypotonic-triggered Ca^2+^ transients in processes before global cytoplasmic Ca^2+^ levels increased. This might reflect Ca^2+^ influx into the confined cytoplasmic volume of the process or local differences in Ca^2+^ regulation. Alternatively, these data could indicate a higher density of osmotically activated channels in these processes.

### Candidate transduction channels in Merkel cells

Several gene families have been implicated in vertebrate mechanotransduction, including members of the TRP family and the DEG/ENaC family. In particular, TRPV4 responds to hypotonic solutions in HEK cells [33], [34] and is expressed in Merkel cells [32]. Somewhat surprisingly, our analysis TRPV4-deficient mice indicates that this channel is not required for swelling-activated Ca^2+^ signals in Merkel cells. Thus, we used pharmacology to elucidate the molecular nature of Merkel cells' hypotonic-activated channel. Amiloride (50 µM), which inhibits most DEG/ENaC channels with an IC_50_ of 0.1–20 µM [47], does not block hypotonic induced Ca^2+^ signals, making these channels unlikely candidates for Merkel-cell transduction channels. Ruthenium red is a broad-spectrum inhibitor that blocks some TRP channels, including TRPV channels. We found that 10 µM ruthenium red inhibits both hypotonic and high-K^+^ induced Ca^2+^ transients in Merkel cells. Since ruthenium red has also been shown to inhibit both VACCs and Ca^2+^-store release in some cell types [48], [49], it is unclear to what extent ruthenium red directly inhibits swelling-activated channels or downstream Ca^2+^ signaling. At any rate, because ruthenium red does not completely block hypotonic-induced Ca^2+^ transients, they cannot be solely mediated by ruthenium-red-sensitive channels, including the TRPV subfamily.

Because other TRP channels play prominent roles in sensory transduction, including mechanotransduction in zebrafish and invertebrates, they remain attractive candidates for investigation. Moreover, many of these channels are not blocked by ruthenium red. We demonstrated with RT-PCR that Merkel cells express robust transcripts encoding six TRP channels, and transcripts were occasionally amplified for an additional five channels. The sporadic amplification of these latter transcripts may indicate that they are present in very low copy number [50].

Among the TRP channels expressed in Merkel cells, TRPC1, PKD1 and PKD2 have been previously implicated in mechanotransduction. Both are Ca^2+^ permeable, and neither is known to be blocked by ruthenium red [36], [51], [52]. Although PKD2 is blocked by amiloride, its IC_50_ is 80 µM, larger than the concentrations than we tested here (see Methods and [53]). Interestingly, PKD1 and PKD2 form Ca^2+^ permeable complexes that transduce fluid flow in the kidney [30]. In human polycystic kidney disease, patients are heterozygous for either PKD1 or PKD2 mutations and suffer from cyst formation and eventual kidney failure [54]. As homozygous deletion in PKD1 or PKD2 in mice causes embryonic death, examination of touch sensitivity in PKD-deficient mice will require a conditional knockout [55]. TRPC1 is activated by membrane tension in CHO- cells and *Xenopus laevis* oocytes [37]; however, the role of TRPC1 *in vivo* is still undetermined [56].

Many of the TRP channels we found expressed in Merkel cells are orphans: little is known of their biophysical properties, let alone their physiological roles in vivo. Consequently, they represent candidate transduction channels. Thus, our findings set the stage for future gene disruption experiments to identify which of these channels mediate swelling-activated signals in Merkel cells in vitro and to determine whether the same channels transduce touch stimuli in vivo.

## Supporting Information

Movie S1 Time series of confocal z-series projections of a Merkel cell during a 20% hypotonic perfusion. The cell surface was visualized with fluorescent microspheres (white spots). Microspheres on the coverslip surface have been removed for clarity. Z-series were collected every 7 s.
(0.43 MB MOV)

Movie S2 Time series of fura-2 fluorescence ratios (F340/F380) show Ca^2+^ increases in Merkel cells during a 20% hypotonic perfusion. A cluster of keratinocytes (right) did not respond to the stimulus. Fura-2 ratios are displayed on a pseudocolor scale. Frames were collected every 2 s.
(1.05 MB MOV)
